# Great Tit (*Parus major*) Uropygial Gland Microbiomes and Their Potential Defensive Roles

**DOI:** 10.3389/fmicb.2020.01735

**Published:** 2020-07-28

**Authors:** Kasun H. Bodawatta, Signe K. Schierbech, Nanna R. Petersen, Katerina Sam, Nick Bos, Knud A. Jønsson, Michael Poulsen

**Affiliations:** ^1^Natural History Museum of Denmark, University of Copenhagen, Copenhagen, Denmark; ^2^Section for Ecology and Evolution, Department of Biology, University of Copenhagen, Copenhagen, Denmark; ^3^Biology Centre of Czech Academy of Sciences, Institute of Entomology, České Budějovice, Czechia; ^4^Faculty of Science, University of South Bohemia, České Budějovice, Czechia

**Keywords:** preen gland, culture-dependent, culture-independent, *Bacillus*, *Kocuria*, defensive symbionts

## Abstract

The uropygial gland (preen gland) of birds plays an important role in maintaining feather integrity and hygiene. Although a few studies have demonstrated potential defensive roles of bacteria residing within these glands, the diversity and functions of the uropygial gland microbiota are largely unknown. Therefore, we investigated the microbiota of great tit (*Parus major*) uropygial glands through both isolation of bacteria (culture-dependent) and 16S rRNA amplicon sequencing (culture-independent). Co-culture experiments of selected bacterial isolates with four known feather-degrading bacteria (*Bacillus licheniformis*, *Kocuria rhizophila*, *Pseudomonas monteilii*, and *Dermacoccus nishinomiyaensis*), two non-feather degrading feather bacteria, one common soil bacterial pathogen and two common fungal pathogens enabled us to evaluate the potential antimicrobial properties of these isolates. Our results show major differences between bacterial communities characterized using culture-dependent and -independent approaches. In the former, we were only able to isolate 12 bacterial genera (dominated by members of the Firmicutes and Actinobacteria), while amplicon sequencing identified 110 bacterial genera (dominated by Firmicutes, Bacteroidetes, and Proteobacteria). Uropygial gland bacterial isolates belonging to the genera *Bacillus* and *Kocuria* were able to suppress the growth of four of the nine tested antagonists, attesting to potential defensive roles. However, these bacterial genera were infrequent in our MiSeq results suggesting that the isolated bacteria may not be obligate gland symbionts. Furthermore, bacterial functional predictions using 16S rRNA sequences also revealed the ability of uropygial gland bacteria to produce secondary metabolites with antimicrobial properties, such as terpenes. Our findings support that uropygial gland bacteria may play a role in feather health and that bacterial symbionts might act as defensive microbes. Future investigations of these bacterial communities, with targeted approaches (e.g., bacterial isolation and chemical analyses), are thus warranted to improve our understanding of the evolution and function of these host-microbe interactions.

## Introduction

Bird feathers provide a plethora of functions varying from flight, insulation and mate attraction (Stettenheim, 1972). Consequently, birds need to spend a significant amount of energy and time on feather maintenance and hygiene (Delius, 1988; Cotgreave and Clayton, 1994). Preening (Delius, 1988), dust bathing (van Liere and Bokma, 1987), and anting (utilization of ants for removal of feather parasites) (Craig, 1999) are all used to keep feathers healthy and free of parasites. Of these feather care strategies, preening is most common, and it includes smearing of secretions from the uropygial (preen) gland onto feathers (Kolattukudy, 1981). The uropygial gland (bilobed in most bird species) is located on the rump of birds (Jacob and Ziswiler, 1982) and is important for plumage health (Møller et al., 2010; Moreno-Rueda, 2010; Jacob et al., 2014) and coloration (Moreno-Rueda, 2016). Uropygial gland secretions aid in keeping feathers waterproof (Kolattukudy, 1981; Jacob and Ziswiler, 1982; Moreno-Rueda, 2017), in olfactory communication (Whittaker et al., 2016; Moreno-Rueda, 2017) and potentially in suppressing microbial antagonists (Shawkey et al., 2003; Vincze et al., 2013; Jacob et al., 2014; Moreno-Rueda, 2017). Although there is contradicting evidence on antimicrobial properties (Moreno-Rueda, 2017; Verea et al., 2017; Jacob et al., 2018), a few studies have documented defensive roles of uropygial gland chemical compounds against feather-degrading bacteria (Shawkey et al., 2003; Reneerkens et al., 2008; Møller et al., 2009; Martín-Vivaldi et al., 2010; Ruiz-Rodríguez et al., 2015; Fülöp et al., 2016; Verea et al., 2017; Braun et al., 2018a).

Uropygial gland secretions are dominated by lipids, mainly esters, that probably provide a protective coating to the feathers (Burger et al., 2004; Haribal et al., 2005, 2009; Montalti et al., 2005; Jacob et al., 2018). Other chemical compounds (non-lipid volatile component) are assumed to be associated with olfactory communication (Hagelin and Jones, 2007; Zhang et al., 2010; Mardon et al., 2011) and potentially antimicrobial defenses (Martín-Vivaldi et al., 2010; Magallanes et al., 2016; Braun et al., 2018a). Overall, these compounds may contribute to the latter by helping to create a physical barrier preventing attachment of pathogens (Reneerkens et al., 2008; Verea et al., 2017; Jacob et al., 2018), facilitating the growth of mutualistic feather bacteria that can outcompete microbial parasites (Shawkey et al., 2003; Møller et al., 2009), and/or through antimicrobial properties (Martín-Vivaldi et al., 2010; Magallanes et al., 2016; Braun et al., 2018a). Our knowledge of antimicrobial compounds within the uropygial secretion remains limited, with only a few predicted potential antimicrobial compounds, such as carboxylic acids (Martín-Vivaldi et al., 2010) and terpenoids (Haribal et al., 2009). One potential source of these antimicrobials could be symbiotic bacteria (Ruiz-Rodríguez et al., 2009, 2012), as demonstrated in *Upupa epops* (Eurasian hoopoe) (Martín-Vivaldi et al., 2010).

Uropygial gland bacteria have been identified for many bird species, mainly through culturing approaches (Law-Brown and Meyers, 2003; Soler et al., 2008; Braun et al., 2016, 2018b,2018c, 2019; Whittaker and Theis, 2016). However, our understanding of the diversity and functions of these bacterial symbionts are limited to a few bird species (Ruiz-Rodríguez et al., 2009; Whittaker et al., 2016; Pearce et al., 2017; Braun et al., 2018a), with a skew toward research in *U. epops* (Martín-Vivaldi et al., 2010, 2018; Ruiz-Rodríguez et al., 2012; Rodríguez-Ruano et al., 2015; Martínez-García et al., 2016). Only a few uropygial microbiome studies have used amplicon sequencing to explore their microbial composition (Martínez-García et al., 2016; Whittaker et al., 2016; Pearce et al., 2017; Rodríguez-Ruano et al., 2018). Uropygial gland microbiomes are generally diverse and dominated by members of the phyla Firmicutes, Proteobacteria, and Actinobacteria, but their relative abundances vary by bird species (Whittaker et al., 2016; Pearce et al., 2017; Rodríguez-Ruano et al., 2018). A limited number of studies have demonstrated that host genetic factors and social interactions shape uropygial gland microbiomes more than do environmental factors (Whittaker et al., 2016; Pearce et al., 2017; Martín-Vivaldi et al., 2018). For example, as seen in both *U. epops* and *Junco hyemalis* (dark-eyed junco), mother to offspring transmission of bacteria is essential for uropygial microbiome composition (Whittaker et al., 2016; Martín-Vivaldi et al., 2018).

Insights into the potential defensive functions of uropygial gland bacterial symbionts are limited to a handful of studies. *Enterococcus faecalis* isolated from *U. epops* was found to inhibit the growth of feather-degrading bacteria (Ruiz-Rodríguez et al., 2009), but *Corynebacterium uropygiale* from *Meleagris gallopavo* (wild turkey) did not reveal such an effect (Braun et al., 2018a). To improve our understanding of the symbioses between birds and the uropygial gland microbiome and their defensive role, we investigated great tit (*Parus major*) uropygial gland microbiomes using three approaches. First, we used a culture-based approach to identify culturable bacteria within the glands. Second, we tested the potential antimicrobial activity of a select set of isolated bacteria, using bioassays against multiple antagonistic microbes, including four feather-degrading bacteria. Third, we explored bacterial community composition within the glands using MiSeq amplicon sequencing of the 16S rRNA. We then used these 16S rRNA MiSeq sequences to predict putative bacterial functions, focusing on metabolic pathways associated with the biosynthesis of antimicrobial compounds that may serve defensive functions.

## Materials and Methods

### Sample Collection

Bacteria were collected from the uropygial glands of 19 captive birds raised in the Czechia (permit OOZP/5345/2018/R La) and five wild birds caught in Denmark (permit J.nr. MST-850-00076) in September 2018. The skin surrounding the gland was cleaned using ethanol and the uropygial gland was massaged until a secretion was released (∼10–20 μL), which was collected using sterile FLOQSwabs^TM^ (Brescia, Italy) and applied directly to Potato Dextrose Agar (PDA, 32 g of PDA mixed with 800 mL of water) medium (a neutral media) containing 50 mg cycloheximide per liter to avoid fungal contamination. Plates were left at room temperature (25°C) in aerobic conditions until colony-forming units (CFUs) emerged. Morphologically different CFUs were subcultured to PDA plates without cycloheximide until pure cultures were obtained. When enough biomass (colony diameter of ∼2 cm) was produced, cultures were harvested for DNA extraction.

Captive birds from the Czechia were euthanized using a carbon dioxide chamber, following the Czechia’s dispensation of the law no. 359/2012 Col., §17, par. 1 (i.e., animal cruelty act) under the permit number MZP/2018/785/1363 issued by the Ministry of the Environment of the Czechia (c.f. Bodawatta et al., 2020). From these individuals, we dissected the uropygial glands and stored them in RNAlater^TM^ in −20°C until DNA extractions for subsequent MiSeq amplicon sequencing of the V4 region of 16S rRNA gene to characterize bacterial community composition.

### DNA Extractions, PCR and Sanger Sequencing on Bacterial Isolates

For bacterial isolates, bacterial biomass was collected using a sterile loop and placed in a 1.5 mL microcentrifuge tube. For whole gland extractions, the gland along with 100 μL RNAlater was used for the DNA extraction. DNA extractions were done using the Qiagen DNeasy Blood and Tissue Kit (Qiagen, Germany) using the manufacture’s guidelines, except for an extended ca. 12 h incubation period at 56°C and the use of heated (40°C) 75 μL AE buffer at the elution step.

PCR on DNA from bacterial isolates were done in 25 μL reactions per sample (8.5 μL water, 12.5 μL VWR Red Taq, 1 μL of each primer and 2 μL DNA template) using primer pair 27F (5′-AGAGTTTGATCCTGGCTCAG-3′) and 1492R (5′-GGTTACCTTGTTACGACTT-3′) targeting the bacterial 16S rRNA gene (Miller et al., 2013). The PCR protocol was as follows: initial denaturing at 94°C for 4 min, 35 cycles of denaturing (94°C for 30 s), annealing (56°C for 30 s), and elongation (72°C for 30 s), followed by final elongation of 4 min at 72°C. After checking PCR products on a 2% agarose gel, products were cleaned using the Stratec MSB Spin PCRapace (Birkenfeld, Germany) cleaning kit. Clean products were Sanger sequenced for both forward and reversed primers at Eurofins Genomics (Ebersberg, Germany).

Sanger sequences of bacterial isolates were merged using Geneious Prime 2019.1.1.^1^ All reverse strands were reverse-complemented and aligned with their corresponding forward strand, using global alignment with free end gaps and the Geneious algorithm with the default settings. Consensus strands were extracted and used for identification of the bacteria using BLASTn on NCBI. Sequence lengths of our isolates varied from 1153–1294 bp, capturing a substantial portion of the 16S rRNA gene (Miller et al., 2013). To generate a phylogeny of the bacterial isolates, we used BEAST v1.8.4 (Drummond et al., 2012), applying the best fitting model of nucleotide evolution (GTR) as determined by the Bayesian Information Criterion (BIC) in jModelTest 2 (Darriba et al., 2012). We included published 16S rRNA sequences from *Kocuria uropygioeca*: MF510150 (isolated from the great spotted woodpecker, *Dendrocopos major*) (Braun et al., 2018b), *Kocuria tytonis*: MG547562 (isolated from the American barn owl, *Tyto furcata*) (Braun et al., 2019), and *E. faecalis*: MT261871 (similar strain isolated from *U. epops*) (Soler et al., 2008) in our phylogenetic tree to investigate the placement of previously isolated uropygial gland bacteria among our isolates. The final analysis was run for 100 million generations using a relaxed uncorrelated lognormal distribution for the molecular clock model, assuming a birth-death speciation process as a tree prior. Convergence diagnostics were assessed in Tracer v1.6 (Rambaut et al., 2014), by determining the effective sample sizes and mean distribution values. The final output tree was summarized in TreeAnnotator v1.8.3 (Drummond et al., 2012) as a maximum clade credibility (MCC) tree after discarding one million generations as burn-in.

### Illumina MiSeq Amplicon Sequencing and Functional Predictions

Initial PCRs on the DNA samples from whole uropygial glands were done using primers SA711 and SB504 (c.f. Bodawatta et al., 2018), with identical PCR conditions as used for the Sanger sequencing of bacterial isolates. DNA from positively amplified samples (10 out of 19 samples) were sent to the Microbial Systems Molecular Biology lab in the University of Michigan for sequencing using an Illumina MiSeq platform. We determined the sex of the birds using PCR with established avian-specific sex primers P2 and P8 (Griffiths et al., 1998; Lezalova-Pialkova, 2011).

**FIGURE 1:**
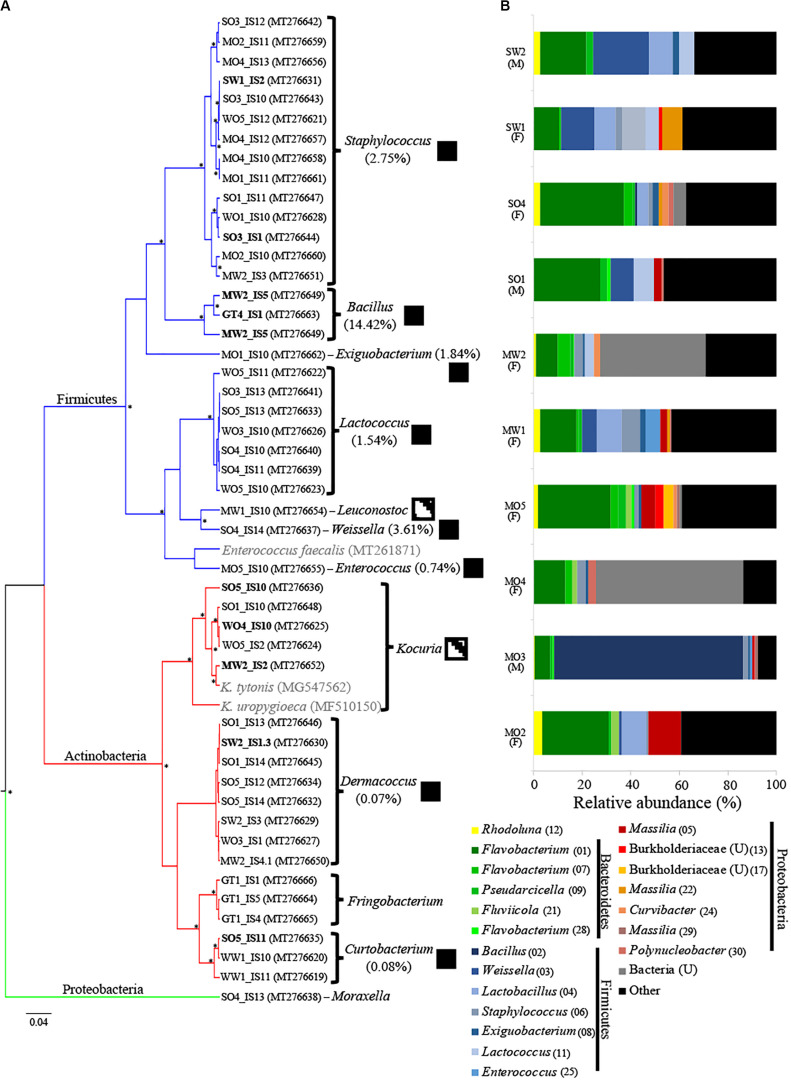
**(A)** Maximum clade credibility tree of bacterial isolates from uropygial glands of *Parus major* (posterior probability values >94% are indicated with asterisks). The GenBank accession numbers of strains are indicated in parentheses. Two *Kocuria* and one *Enterococcus* strains previously isolated from the uropygial glands of other bird species are shown in gray. Branch color corresponds to bacterial phyla of the isolates. Isolates that were used in the co-culture experiment are shown in bold. Boxes to the right of genus names indicate the presence of bacterial genera (black) or families (dashed boxes) among the 16S rRNA MiSeq analysis of whole uropygial glands, with the percentages indicating relative abundance of each genus in the MiSeq data. **(B)** Relative abundance of the top 20 ASVs that were identified to at least family level in the MiSeq analysis. Each bar represents an individual uropygial gland and sex of the bird is shown in parenthesis below the code name of the individual (M: male and F: female). Unidentified genera are indicated with a “U” and ASV numbers are given in parentheses.

Amplicon sequences were analyzed using the DADA2 (Callahan et al., 2016) pipeline within QIIME2 (Bolyen et al., 2019), and sequences were aligned using the Silva132 (Quast et al., 2013) bacterial reference library. Sequences were identified to amplicon sequence variances (ASVs) at 100% similarity and archaeal, mitochondrial, and chloroplast sequences were removed. ASVs with less than 10 sequences and samples with less than 750 sequences were removed from further analyses. All downstream analyses were done in R (R Core Team, 2019). We conducted permutational multivariate analysis of variance (PERMANOVA) to investigate the uropygial microbial community-level differences between females and males (sexes can be found in Supplementary Table S1 and Figure 1) using the vegan package (Oksanen et al., 2019). Furthermore, we investigated bacterial ASVs with relative abundance of >0.1% in >50% of samples utilizing the microbiome (Lahti and Shetty, 2017) and phyloseq (McMurdie and Holmes, 2013) packages.

Predicted functional capabilities of uropygial gland bacteria were explored through metagenomic predictions based on the 16S rRNA MiSeq sequences using PICRUSt2 (Douglas et al., 2019). We investigated the predicted microbial metabolic pathways using the MetaCyc database (Caspi et al., 2006) to identify metabolic pathways associated with antimicrobial defenses. We only focused on metabolic pathways that were predicted in more than 80% of the samples to investigate common microbial functions. PICRUSt2 analysis was only used to investigate the potential metabolic pathways and results should be interpreted with caution, as they only represent predictions based on insights from available genomes.

### Bioassays of Antimicrobial Activity of Bacterial Isolates

Antimicrobial activities of a subset of our isolated strains (three *Bacillus*, three *Kocuria*, one *Dermacoccus*, one *Curtobacterium*, and two *Staphylococcus*) were tested (Table 1). We did not use isolates of the other seven genera due to their slow growth rates and low frequencies (five genera only had one isolate). Bioassays were done in aerobic conditions to mimic the feather environment. This was mainly due to growth conditions of antagonists used in this experiment (Shawkey et al., 2003) and to avoid potential false reduced growth of antagonists under anaerobic conditions. Conducting bioassays under aerobic conditions at room temperature is further justified by the non-detection of antimicrobial compounds in a previous study on *P. major* uropygial gland secretions (Jacob et al., 2018), and by the potential of birds to apply bacteria directly onto their feathers to suppress antagonistic microbes. Biomass from bacterial isolates were mixed in 1 mL 1× Phosphate-Buffered Solution (PBS; 8 g NaCl, 0.2 g KCl, 1.44 g Na_2_HPO_4_.2H_2_O, 0.24 g KH_2_PO, and 800 mL distilled water) to acquire a homogenous cell suspension. From this homogenous mix, 10 μL was point inoculated onto 100 new PDA plates per isolate to introduce identical numbers of starting bacterial cells. After one week, we introduced 10 μL (mixed in 1 mL of 1× PBS) each microbial antagonist to 10 plates of each bacterial isolate, generating 10 replicates per combination. The inoculum of antagonist was introduced approximately 1 cm away from the colonies of the uropygial bacterial isolates. Some of the replicates were lost due to fungal contaminations (but 67 out of 90 combinations had four or more replicates: see details below). The tested antagonists were four feather-degrading bacteria (*Bacillus licheniformis* - DSM13, *Kocuria rhizophila* - DSM 11926, *Pseudomonas monteilii* - DSM 1388, and *Dermacoccus nishinomiyaensis* - DSM 105516) (Shawkey et al., 2003), two non-feather-degrading bacteria isolated from bird feathers (*Bacillus thuringiensis* - DSM 104061 and *Staphylococcus epidermidis* - DSM 103867) (Shawkey et al., 2003), one common pathogenic bacterium (*Pseudomonas aeruginosa*), one filamentous fungus (*Aspergillus niger*), and one yeast (*Candida catenulata* - DSM 70040). Pictures of plates were taken on a black background every third or fourth day for four weeks starting 24 h after the introduction of the antagonists. As controls, we grew bacterial isolates and pathogens alone (see Supplementary Figure S1 for representative images of bacteria-target interactions).

**TABLE 1:** Uropygial gland bacterial isolates that were used in the co-culture experiment and their closest relatives identified from GenBank.

**Isolate**	**Closest relative in GenBank**	**GenBank accession number of the closest strain**	**Sequence similarity (%)**
MW2_IS1	*Bacillus subtilis*	NR_112116.2	99.9
MW2_IS5	*Bacillus aerius*	NR_042338.1	99.9
GT4_IS1	*Bacillus pumilus*	NR_043242.1	99.6
SO3_IS1	*Staphylococcus haemolyticus*	NR_036955.1	99.7
SW1_IS2	*Staphylococcus pasteuri*	NR_024669.1	99.8
SO5_IS10	*Kocuria marina*	NR_025723.1	99.6
MW2_IS2	*Kocuria rhizophila*	NR_026452.1	99.9
WO4_IS10	*Kocuria salsiccia*	NR_117299.1	99.9
SO5_IS10	*Curtobacterium flaccumfaciens*	NR_025467.1	99.9
SW2_IS1.3	*Dermacoccus profundi*	NR_043262	99.8

The growth area of each strain (both isolates and antagonists, in co-culture and alone) per plate was estimated by averaging the shortest and longest diameter of colonies using ImageJ (Schneider et al., 2012). Area measurements were subsequently used for two analyses: (1) the effect of bacterial isolates on the growth of antagonists; (2) the effect of antagonists on the growth of bacterial isolates. As the data violated assumptions of parametric models, we used Kruskal–Wallis tests (dplyr package; Wickham et al., 2019) followed by Dunnett’s *post hoc* test, in order to compare the growth of a strain in competition to its growth alone (multcomp package; Hothorn et al., 2008).

## Results

### Bacterial Isolates From the Uropygial Gland Secretions

We isolated 48 bacterial isolates from the uropygial gland secretions of *P. major* in aerobic conditions (Figure 1A). We were only able to culture bacteria from 19 out of the 24 bird individuals, with an average of 2.53 (±0.327 SE) isolates per individual. Firmicutes were represented by seven genera, including *Staphylococcus* (14 isolates), *Lactococcus* (7), *Bacillus* (3), *Weissella* (1), *Leuconosto*c (1), *Enterococcus* (1), and *Exiguobacterium* (1). Actinobacteria included four genera, including *Dermacoccus* (8 isolates), *Frigoribacterium* (3), *Curtobacterium* (3), and *Kocuria* (5) (Figure 1A and Supplementary Table S1).

### Culture-Independent Inventories of Whole Uropygial Microbial Communities

After quality filtering, we retained 18,152 sequences [1895.5 ± 418.9 (SE) per sample] that were classified into 253 ASVs (Supplementary Table S2). We observed high variation in microbiome composition between individual birds (Figure 1B), but gland microbiomes were on average dominated by Firmicutes (31.2%), followed by Bacteroidetes (28.1%), Proteobacteria (20.2%), and Actinobacteria (3.5%), while 10.8% of the sequences could not be classified at the phylum level (Figure 1B). The 20 most abundant ASVs (including only ASVs that were identified to at least family level) represented 59.1% of all sequences (Figure 1B) and the majority of these were within the bacterial orders Flavobacteriales (Bacteroidetes), Bacillales (Firmicutes), Lactobacillales (Firmicutes), and Burkholderiales (Proteobacteria) (Figure 1B). Males harbored lower ASV richness (mean ± SE: 28.67 ± 1.2) in their uropygial glands than females (42.71 ± 9.8), but this was not significantly different (Kruskal–Wallis: *H* = 2.208, *df* = 1, *p* = 0.1373). The microbial community structure did also not differ between the sexes (PERMANOVA_10_,_000_ _permutations_: *F* = 0.9328, *R*^2^ = 0.1044, *p* = 0.5981). However, this may be an artifact of biased sampling of sexes (seven females and three males) in our data set (Figure 1B and Supplementary Table S2).

There were only seven ASVs that were frequently present in uropygial microbiomes (relative abundance >0.1% in >50% of samples). These ASVs included the genera *Flavobacterium* (ASV 1 and 7), *Weissella* (ASV 3), *Staphylococcus* (ASV 6), *Exiguobacterium* (ASV 8), *Pseudarcicella* (ASV 9), and *Rhodoluna* (ASV 12). We were able to isolate strains belonging to three of these genera (*Weissella*, *Staphylococcus*, and *Exiguobacterium*). Overall, however, we were only able to isolate 7.3% (8 of 110) of the genera identified using MiSeq (Figure 1).

### Microbial Metabolic Pathways and Functional Predictions

**FIGURE 2:**
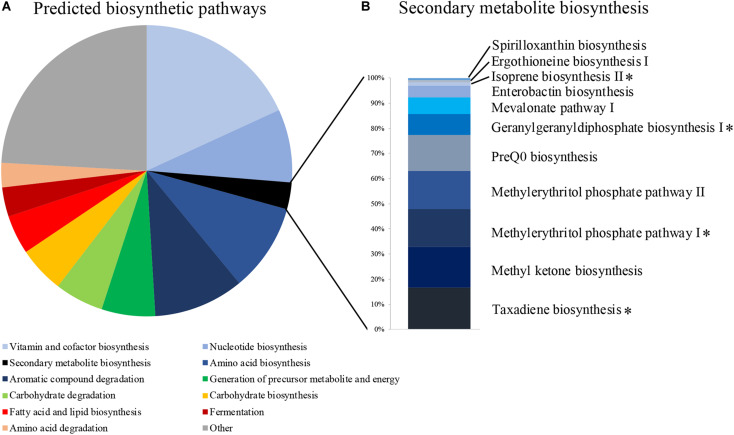
**(A)** The proportion of predicted metabolic pathways using the MetaCyc database based on all bacterial 16S rRNA sequences of uropygial gland microbiomes. **(B)** Relative abundances of the 11 pathways that are associated with secondary metabolite biosynthesis. Pathways associated with terpene biosynthesis are highlighted with asterisks.

We identified 471 predicted microbial metabolic pathways, 370 of which were found in more than 80% of our samples (Supplementary Table S3). The majority of these pathways were associated with functions related to cell maintenance, such as vitamin and cofactor biosynthesis (67 pathways), amino acid biosynthesis (36), aromatic compound degradation (37), nucleotide biosynthesis (30), and carbohydrate degradation (20) (Figure 2A). Eleven pathways were associated with secondary metabolite production (Figure 2B and Supplementary Table S3), of which four were associated with diterpene and hemiterpene biosynthesis and one with the biosynthesis of carotenoids. Multiple bacterial genera, including *Bacillus*, have the potential to produce these terpenoids (Supplementary Table S4).

### Bioassays of Antimicrobial Activity of Bacterial Isolates

**FIGURE 3:**
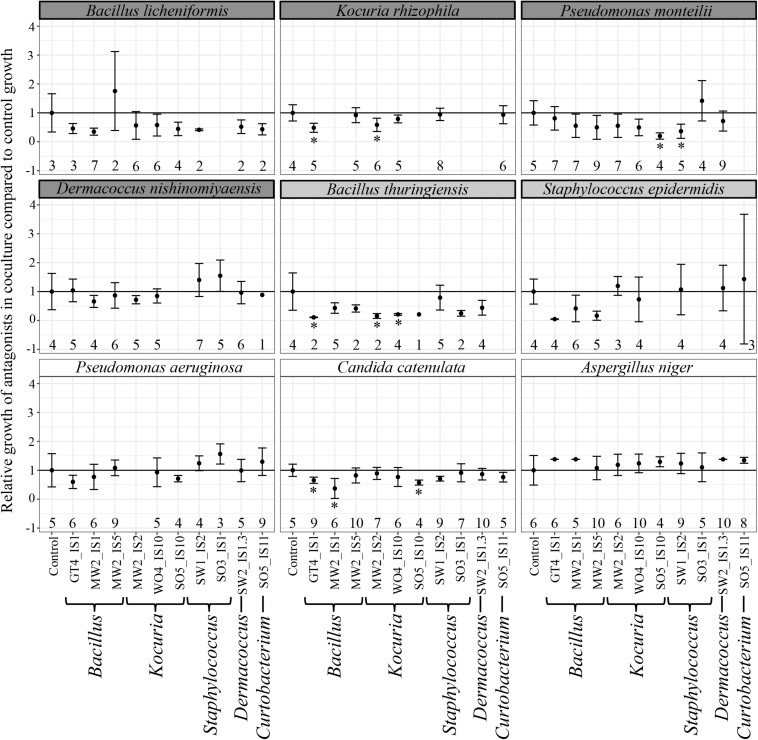
Relative growth (±SE) of antagonists with different bacterial isolates compared to control growth, with names of feather-degrading bacteria having a dark gray background, and names of non-feather degrading bacteria isolated from feathers with a light gray background. Growth that was significantly reduced compared to controls is indicated with an asterisk. The number of replicates is indicated below each data point. Relative growth was calculated as the growth of a strain in a sample divided by the average growth of its respective control.

The majority of antagonists (Supplementary Figure S2) and bacterial isolates (Supplementary Figure S3) reached stable growth after 28 days (4 weeks), indicating that the duration of our experiment was sufficient to test for effects on growth. Due to fungal infections, we lost ca. 50% of our cultures, leading to smaller sample sizes than initially planned. However, we had sufficient replicates to perform statistical analyses and observed significant impacts on growth of multiple antagonist by bacterial isolates (Table 2). Pairwise comparisons only revealed significant growth suppression of four antagonists by few bacterial isolates (Figure 3, Supplementary Figure S4, and Supplementary Table S5). However, many bacterial isolates still demonstrated the tendency to suppress the growth of many tested antagonists (Figure 3). Bacterial strain GT4_IS1 (closely related to *Bacillus pumilus*) (Table 1) significantly reduced the growth of three antagonists, while strains MW2_IS2 (closely related to *K. rhizophila*) and SO5_IS10 (closely related to *Kocuria marina*) (Table 1) significantly reduced the growth of two antagonists each (Figure 3 and Supplementary Figure S4). Growth of two out of four feather-degrading bacteria (except *B. licheniformis* and *D. nishinomiyaensis*) were restricted by at least one of our bacterial isolates in the genera *Bacillus*, *Kocuria*, and *Staphylococcus* (Figure 3). However, we still observed growth reduction in *B. licheniformis* in co-culture with *Bacillus* strains. Comparison of the growth of bacterial isolates with and without antagonists yielded an overall significant and negative effect of antagonist presence (Supplementary Table S6 and Supplementary Figure S5). However, this was due to inhibition of most bacterial isolates by *A. niger* (Supplementary Figure S3) and excluding this fungus from the analysis removed the effect in most cases, and particularly for *Bacillus* and *Kocuria* isolates (Supplementary Table S6).

**TABLE 2:** Results of the Kruskal–Wallis rank-based non-parametric analysis of growth of antagonists with and without different uropygial gland bacterial isolates.

**Antagonist**	**Antagonist type**	**H**	**df**	***p***
*Bacillus licheniformis*	Feather degrading	9.986	9	0.3516
*Kocuria rhizophila*	Feather degrading	17.02	6	**0.0092**
*Pseudomonas monteilii*	Feather degrading	21.51	9	**0.0106**
*Dermacoccus nishinomiyaensis*	Feather degrading	19.25	9	**0.0232**
*Bacillus thuringiensis*	Feather isolate	18.35	9	**0.0313**
*Staphylococcus epidermidis*	Feather isolate	18.56	8	**0.0174**
*Pseudomonas aeruginosa*	Soil bacteria	20.06	9	**0.0175**
*Aspergillus niger*	Filamentous soil fungus	10.28	10	0.4164
*Candida catenulata*	Yeast	21.38	10	**0.0186**

*Dunnett’s *post hoc* tests results are given in Supplementary Table S4. Significant *p*-values are given in bold.*

## Discussion

To improve our understanding of uropygial gland microbiomes and their proposed defensive functions against microbial antagonists, we explored *P. major* uropygial gland microbiomes through culture-dependent and -independent methods and tested for putative antimicrobial properties of a set of uropygial gland bacterial isolates. Aligning with other animal microbiome studies (Creevey et al., 2014; Fenske et al., 2020), we were only able to isolate and culture a small fraction of the gland microbiome (Figure 1). MiSeq amplicon sequencing revealed a diverse uropygial gland microbiome with representation of the bacterial phyla Firmicutes, Bacteroidetes, and Proteobacteria, and we found high individual variation in these microbiomes, comparable to what has been found in other uropygial microbiome studies (Pearce et al., 2017; Rodríguez-Ruano et al., 2018). However, the major bacterial groups in *P. major* differ from uropygial gland microbiomes of *U. epops* (Rodríguez-Ruano et al., 2018) and *Oceanodroma leucorhoa* (Leach’s storm petrel) (Pearce et al., 2017), as we find relatively more Firmicutes and Bacteroidetes but relatively few Proteobacteria. This supports potential species specificity in the structure of these microbiomes. The composition of the uropygial microbiome differed from that of the digestive tract microbiome of *P. major* (Bodawatta et al., 2020), attesting to the unique microbiome of this gland. The bacterial genera we were able to isolate allowed us for the first time to test for potential defensive properties of *P. major* uropygial gland bacteria. A few of the bacterial isolates restricted the growth of feather-degrading bacteria, supporting the defensive potential observed in bacteria isolated from the *U. epops* uropygial glands (Martín-Vivaldi et al., 2010; Soler et al., 2010). Metagenomic predictions based on 16S rRNA sequences demonstrated the potential for uropygial gland bacteria to produce antimicrobial secondary metabolites such as terpenes (Yamada et al., 2015), which may aid in their defensive role against antagonistic microorganisms.

The bacteria isolated from the uropygial glands of *P. major* represented multiple genera, including *Kocuria* and *Enterococcus*, which have also been isolated from uropygial glands of other bird species (Soler et al., 2008; Braun et al., 2018b, 2019). Although an *Enterococcus* strain isolated from *U. epops* demonstrated antimicrobial activity against feather-degrading bacteria (Soler et al., 2010), we were unable to test this with our strain due to its slow growth in laboratory conditions and our inability to acquire enough replicates. We managed to isolate three genera (*Weissella*, *Staphylococcus*, and *Exiguobacterium*) of the seven most frequent bacterial ASVs found in the MiSeq sequencing, suggesting that our isolation techniques indeed capture persistent bacterial genera of *P. major* uropygial glands. However, we only tested the effect of two *Staphylococcus* strains against antagonistic microbes and, except for growth restriction of *P. monteilii* by the isolate SW1_IS2, we did not see an impact of *Staphylococcus* on the antagonists. The limitations in being able to culture other bacteria that are present in the MiSeq data, and that have previously been isolated from uropygial glands of other birds, such as *Corynebacterium* (Braun et al., 2016; Braun et al., 2018c), thus limited a fuller exploration of potential defensive roles across microbiome members.

*Bacillus* isolates exhibited the strongest and broadest growth suppression of antagonists (Figure 3), suggesting that this genus is promising for further exploration as a defensive symbiont. Members of this genus have also been suggested to play a defensive role in fungus-growing termites (Um et al., 2013), leafhoppers (Sivakumar et al., 2017), and maize (Gond et al., 2015). *Bacillus* isolates GT4_IS1 and MW2_IS1 were closely related to the well-known antibiotic producers *B. pumilus* and *Bacillus subtilis*, respectively (Leifert et al., 1995; Stein, 2005; Kovács, 2019), suggesting potential defensive roles. However, our amplicon sequencing revealed that *Bacillus* is generally infrequent in uropygial glands (Figure 1B), which suggests that it is unlikely to be a common defensive symbiont in *P. major*. Consequently, future studies should focus on isolating more common bacterial genera using more specialized media and growth conditions to obtain members of the uncultured fraction of the uropygial gland microbiome. None of our bacterial isolates impacted the growth of all tested antagonists, but collectively (particularly *Bacillus* and *Kocuria*) restricted the growth of four out of nine antagonists. The consortium of bacterial symbionts may thus collectively reduce the microbial pathogen load on bird feathers, but members of this consortium can vary between individuals.

Our metagenomic predictions based on the 16S rRNA amplicons detected four secondary metabolite biosynthetic pathways associated with the production of terpenes (Figure 2B). Terpenes are known to inhibit many bacterial pathogens (Greay and Hammer, 2011). Many of the common bacterial genera (e.g., *Rhodoluna*, *Flavobacterium*, *Massilia*, *Staphylococcus*, *Lactobacillus*) observed in our MiSeq data and some genera of our bacterial isolates (i.e., *Bacillus* and *Staphylococcus*) are also capable of producing these terpenoids (Supplementary Table S4; Yamada et al., 2015). Terpene derivatives have also been found frequently in uropygial glands of neotropical birds, suggesting that they may play an important role to their hosts, possibly associated with defense (Haribal et al., 2009). However, a previous study investigating the chemistry of *P. major* uropygial gland secretions did not find any antimicrobials (Jacob et al., 2018). Instead, Jacob et al. (2018) hypothesized that *P. major* use these secretions directly on the feathers to create a physical barrier, which then reduces colonization of pathogenic microbes and/or maintain symbiotic feather bacteria that out-compete microbial pathogens. Our results suggest that uropygial bacteria with potential antimicrobial properties could be applied onto the feathers along with uropygial gland secretions, potentially contributing to inhibiting growth of antagonistic microbes upon contact. However, chemical analysis on secretions from different uropygial gland bacterial isolates are needed to pinpoint the potential defense mechanisms of these symbionts. The uropygial gland microbiomes also contained many bacterial genera that were found in feathers of other bird species (Whittaker and Theis, 2016; Whittaker et al., 2016; van Veelen et al., 2017; Javurková et al., 2019), suggesting that uropygial glands might act as reservoirs of feather microbes. Furthermore, the presence of odor-producing bacteria, such as *Burkholderia* and *Pseudomonas* in *P. major* uropygial microbiomes (Whittaker and Theis, 2016; Whittaker et al., 2016), supports a potential association of uropygial gland microbiomes with bird olfactory communication (Whittaker and Theis, 2016; Maraci et al., 2018).

We acknowledge that our study is an initial step toward exploring the uropygial gland microbiomes, and many improvements can be done in future studies to help elucidate the diversity and function of these microbiomes. The culturable portion of the microbiome can be improved through homogenous application (mixing them in a buffer before introducing them to growth media) of uropygial secretions in different growth media (Braun et al., 2016) and incubation under conditions that better mimic the uropygial gland environment (e.g., lower oxygen and higher temperature). The functions of bacterial isolates can naturally also be more firmly predicted through metagenomics and metatranscriptomics, coupled with antimicrobial assays using bacterial chemical extract tests (cf. Um et al., 2013; Kildgaard et al., 2018). The use of chemical extracts would enable testing of the potential effects of uropygial microbes against antagonists growing in aerobic conditions without introducing any growth biases (i.e., sub-optimal conditions for symbiotic bacteria), inherently associated with our co-culturing approach. Furthermore, the use of agar with keratinases to mimic environments with feather-degrading bacteria may also be a promising alternative to co-culturing (Braun et al., 2018a). After identifying consistent and putative defensive symbionts of uropygial glands, evaluation of densities of these targeted taxa within uropygial glands, using methods such as droplet digital PCR (ddPCR) and fluorescence *in situ* hybridization (FISH) (Davidson and Stahl, 2008; Sanders et al., 2017; Zhukova et al., 2017), could further help localize and validate their potential contributions to defense. We thus advocate for integrative approaches that combine microbiological, -omics and chemical analyses methods to further improve our knowledge of potential defensive functions of uropygial gland microbiomes of birds.

The uropygial gland is an important organ that facilitates feather health and integrity (Kolattukudy, 1981; Shawkey et al., 2003; Vincze et al., 2013; Jacob et al., 2014, 2018; Moreno-Rueda, 2017). We have demonstrated the usefulness of combining culture-dependent and -independent methods to investigate the diversity and potential functions of uropygial gland bacteria of *P. major*. Discrepancies in our approaches further attest to the importance of examining microbiomes with multiple independent methods. To improve our understanding of the evolution and long-term associations in this host-microbial symbioses, investigation of microbiomes across multiple host species across space (e.g., populations) and time (e.g., breeding vs. non-breading seasons), are particularly warranted.

## Data Availability Statement

The datasets presented in this study can be found in online repositories [SRA archive at NCBI (MiSeq data: SAMN14479100–SAMN14479109) and GenBank (Sanger sequences: MT276619–MT276666)] and in the article/Supplementary Material.

## Ethics Statement

The animal study was reviewed and approved by the Denmark Ringing Centre (Permit J.nr. MST-850-00076) and Ministry of the Environment of the Czech Republic (Permits OOZP/5345/2018/R La and MZP/2018/785/1363).

## Author Contributions

KB, KS, KJ, and MP developed the idea and collected the samples. KB, SS, and NP conducted the experiments, lab work, and bioinformatic analysis. KB and NB conducted statistical analysis. All authors commented on manuscript drafts.

## Conflict of Interest

The authors declare that the research was conducted in the absence of any commercial or financial relationships that could be construed as a potential conflict of interest.

## Footnotes

1 www.geneious.com
