# Supplementary material for: Great Tit (Parus major) Uropygial Gland Microbiomes and Their Potential Defensive Roles
Source: Front Microbiol. 2020 Jul 28;11:1735. doi: 10.3389/fmicb.2020.01735 (PMC7401573; doi:10.3389/fmicb.2020.01735)
Supplement: Supplementary file 4 [file Table_4.docx]

**Supplementary material**

**Great tit (*Parus major*) uropygial gland microbiomes and their potential defensive roles**

Kasun H. Bodawatta^1*^, Signe K. Schierbech^1,2^, Nanna R. Petersen^1,2^, Katerina Sam^3, 4^, Nick Bos^2^, Knud A. Jønsson^1^ and Michael Poulsen^2^

# ^1^ Natural History Museum of Denmark, University of Copenhagen, Copenhagen, Denmark

^2^ Section for Ecology and Evolution, Department of Biology, University of Copenhagen, Copenhagen, Denmark

^3^ Biology Centre of Czech Academy of Sciences, Institute of Entomology, Ceske Budejovice, Branisovska 31, Czech Republic

^4^ Faculty of Science, University of South Bohemia, Ceske Budejovice, Branisovska 1760, Czech Republic

*Correspondence

Corresponding Author

[bodawatta@snm.ku.dk](mailto:bodawatta@snm.ku.dk), +45 91724996

**Supplementary Tables**

Table S1. Bacterial isolates from *Parus major* uropygial glands, their closest matches and accession numbers in GenBank (separate Excel file).

Table S2. Amplicon sequence variant (ASV) table of uropygial gland microbiomes of *Parus major*. GenBank accession numbers are given with the respective individual (separate Excel file).

Table S3. Bacterial metabolic pathways predictions assigned using the MetaCyc database based on the 16s rRNA amplicon sequences (separate Excel file).

Table S4. Four predicted (based on 16S rRNA sequences) metabolic pathways associated with biosynthesis of terpenes and their associated major bacterial genera (ASVs that accounted for more than 1% of each pathway abundance).

| Pathway | Total pathway abundance | Number of ASVs predicted to have the pathway | Bacterial genera accounted for major proportion of pathway abundance |
| --- | --- | --- | --- |
| Taxadiene biosynthesis | 6701 | 326 | *Rhodoluna* (Actinobacteria)  *Fluviicola* (Bacteroidetes)  *Flavobacterium* (Bacteroidetes)  *Pseudarcicella* (Bacteroidetes)  Burkholderiaceae_U (Proteobacteria)  *Massilia* (Proteobacteria)  *Curvibacter* (Proteobacteria)  *Bacillus* (Firmicutes)  *Weissella* (Firmicutes)  *Staphylococcus* (Firmicutes)  *Lactobacillus* (Firmicutes) |
| Methylerythritol phosphate pathway 1 | 6230 | 326 | *Rhodoluna* (Actinobacteria)  *Fluviicola* (Bacteroidetes)  *Flavobacterium* (Bacteroidetes)  Burkholderiaceae_U (Proteobacteria)  *Massilia* (Proteobacteria)  *Methylobacterium* (Proteobacteria)  *Polynucleobacter* (Proteobacteria)  *Bacillus* (Firmicutes)  *Weissella* (Firmicutes)  *Staphylococcus* (Firmicutes) |
| Geranylgeranyldiphosphate biosynthesis 1 (via mevalonate) | 4034 | 326 | *Rhodoluna* (Actinobacteria)  *Flavobacterium* (Bacteroidetes)  *Pseudarcicella* (Bacteroidetes)  Burkholderiaceae_U (Proteobacteria)  *Bacillus* (Firmicutes)  *Weissella* (Firmicutes)  *Staphylococcus* (Firmicutes)  *Lactobacillus* (Firmicutes)  *Lactococcus* (Firmicutes) |
| Isoprene biosynthesis 2 | 39 | 35 | *Rhodoluna* (Actinobacteria)  *Flavobacterium* (Bacteroidetes)  *Pseudarcicella* (Bacteroidetes)  *Chryseobacterium* (Bacteroidetes)  Burkholderiaceae_U (Proteobacteria)  *Comamonas* (Proteobacteria)  *Hydrogenophaga* (Proteobacteria)  *Sphingorhabdus* (Proteobacteria)  *Weissella* (Firmicutes)  *Lactobacillus* (Firmicutes)  *Lactococcus* (Firmicutes) |

Table S5. Pair-wise comparisons (Dunnett’s post hoc test) between growth of antagonists on day 28. Results are only shown for control and growth with uropygial bacterial isolates. Significantly different comparisons are indicated in bold.

| Antagonist | Comparison | t value | p |
| --- | --- | --- | --- |
| *Bacillus licheniformis* | Control vs. GT4_IS1 | -1.540 | 0.544 |
|  | Control vs. MW2_IS1 | -2.189 | 0.197 |
|  | Control vs. MW2_IS5 | 1.921 | 0.313 |
|  | Control vs. MW2_IS2 | -1.426 | 0.624 |
|  | Control vs. WO4_IS10 | -1.389 | 0.650 |
|  | Control vs. SO5_IS10 | -1.686 | 0.448 |
|  | Control vs. SW1_IS2 | -1.490 | 0.579 |
|  | Control vs. SW2_IS1.3 | -1.220 | 0.767 |
|  | Control vs. SO5_IS11 | -1.446 | 0.610 |
| *Kocuria rhizophila* | Control vs. GT4_IS1 | -3.291 | **0.011** |
|  | Control vs. MW2_IS5 | -0.507 | 0.986 |
|  | Control vs. MW2_IS2 | -2.748 | **0.042** |
|  | Control vs. WO4_IS10 | -1.343 | 0.547 |
|  | Control vs. SW1_IS2 | -0.365 | 0.998 |
|  | Control vs. SO5_IS11 | -0.430 | 0.994 |
| *Pseudomonas monteilii* | Control vs. GT4_IS1 | -0.825 | 0.957 |
|  | Control vs. MW2_IS1 | -1.945 | 0.276 |
|  | Control vs. MW2_IS5 | -2.296 | 0.141 |
|  | Control vs. MW2_IS2 | -1.945 | 0.276 |
|  | Control vs. WO4_IS10 | -2.124 | 1.984 |
|  | Control vs. SO5_IS10 | -3.050 | **0.024** |
|  | Control vs. SW1_IS2 | -2.563 | **0.079** |
|  | Control vs. SO3_IS1 | 1.593 | 0.483 |
|  | Control vs. SW2_IS1.3 | -1.297 | 0.697 |
| *Dermacoccus nishinomiyaensis* | Control vs. GT4_IS1 | 0.151 | 1.000 |
|  | Control vs. MW2_IS1 | -1.415 | 0.641 |
|  | Control vs. MW2_IS5 | -0.538 | 0.998 |
|  | Control vs. MW2_IS2 | -1.051 | 0.879 |
|  | Control vs. WO4_IS10 | -0.564 | 0.997 |
|  | Control vs. SW1_IS2 | 1.618 | 0.496 |
|  | Control vs. SO3_IS1 | 2.048 | 0.248 |
|  | Control vs. SW2_IS1.3 | -0.141 | 1.000 |
|  | Control vs. SO5_IS11 | -0.251 | 1.000 |
| *Bacillus thuringiensis* | Control vs. GT4_IS1 | -3.078 | **0.041** |
|  | Control vs. MW2_IS1 | -2.538 | 0.122 |
|  | Control vs. MW2_IS5 | -2.018 | 0.309 |
|  | Control vs. MW2_IS2 | -2.913 | **0.051** |
|  | Control vs. WO4_IS10 | -3.339 | **0.023** |
|  | Control vs. SO5_IS10 | -2.111 | 0.266 |
|  | Control vs. SW1_IS2 | -0.934 | 0.945 |
|  | Control vs. SO3_IS1 | -2.598 | 0.109 |
|  | Control vs. SW2_IS1.3 | -2.366 | 0.169 |
| *Staphylococcus epidermidis* | Control vs. GT4_IS1 | -1.684 | 0.431 |
|  | Control vs. MW2_IS1 | -1.131 | 0.806 |
|  | Control vs. MW2_IS5 | -1.555 | 0.515 |
|  | Control vs. MW2_IS2 | 0.321 | 1.000 |
|  | Control vs. WO4_IS10 | -0.479 | 0.998 |
|  | Control vs. SW1_IS2 | 0.123 | 1.000 |
|  | Control vs. SW2_IS1.3 | 0.217 | 1.000 |
|  | Control vs. SO5_IS11 | 0.705 | 0.979 |
| *Pseudomonas aeruginosa* | Control vs. GT4_IS1 | -0.761 | 0.413 |
|  | Control vs. MW2_IS1 | -1.005 | 0.910 |
|  | Control vs. MW2_IS5 | 0.402 | 1.000 |
|  | Control vs. WO4_IS10 | -0.293 | 1.000 |
|  | Control vs. SO5_IS10 | -1.136 | 0.843 |
|  | Control vs. SW1_IS2 | 0.952 | 0.932 |
|  | Control vs. SO3_IS1 | 2.020 | 0.267 |
|  | Control vs. SW2_IS1.3 | -0.037 | 1.000 |
|  | Control vs. SO5_IS11 | 1.423 | 0.649 |
| *Aspergillus niger* | Control vs. GT4_IS1 | 0.296 | 1.000 |
|  | Control vs. MW2_IS1 | 0.284 | 1.000 |
|  | Control vs. MW2_IS5 | -1.460 | 0.546 |
|  | Control vs. MW2_IS2 | -0.745 | 0.972 |
|  | Control vs. WO4_IS10 | -0.518 | 0.998 |
|  | Control vs. SO5_IS10 | -0.155 | 1.000 |
|  | Control vs. SW1_IS2 | -0.523 | 0.998 |
|  | Control vs. SO3_IS1 | -1.209 | 0.730 |
|  | Control vs. SW2_IS1.3 | 0.323 | 1.000 |
|  | Control vs. SO5_IS11 | 0.108 | 1.000 |
| *Candida catenulata* | Control vs. GT4_IS1 | -2.864 | **0.044** |
|  | Control vs. MW2_IS1 | -4.254 | **<0.001** |
|  | Control vs. MW2_IS5 | -1.485 | 0.626 |
|  | Control vs. MW2_IS2 | -0.851 | 0.971 |
|  | Control vs. WO4_IS10 | -1.757 | 0.433 |
|  | Control vs. SO5_IS10 | -2.880 | **0.042** |
|  | Control vs. SW1_IS2 | -1.759 | 0.079 |
|  | Control vs. SO3_IS1 | -0.978 | 0.328 |
|  | Control vs. SW2_IS1.3 | -0.954 | 0.339 |
|  | Control vs. SO5_IS11 | -1.084 | 0.278 |

Table S6. Results of Kruskal-Wallis rank based (non-parametric) analysis on growth of bacterial isolates with and without different microbial antagonists. The results of the Kruskal-Wallis test after removal of *Aspergillus niger* is shown within parenthesis; significant p values in bold.

| Bacterial strain | Genus | H | df | p |
| --- | --- | --- | --- | --- |
| GT4_IS1 | *Bacillus* | 18.70 (14.45) | 9 (8) | **0.0279** (0.0707) |
| MW2_IS1 | *Bacillus* | 21.37 (12.18) | 8 (7) | **0.0062** (0.0947) |
| MW2_IS5 | *Bacillus* | 23.30 (8.863) | 9 (8) | **0.0056** (0.354) |
| MW2_IS2 | *Kocuria* | 13.27 (9.851) | 8 (7) | 0.1031 (0.1972) |
| WO4_IS10 | *Kocuria* | 28.72 (7.003) | 9 (8) | **0.0007** (0.5364) |
| SO5_IS10 | *Kocuria* | 5.977 (4.274) | 6 (5) | 0.4258 (0.5107) |
| SW1_IS2 | *Staphylococcus* | 40.47 (26.73) | 9 (8) | **>0.0001 (0.0008)** |
| SO3_IS1 | *Staphylococcus* | 17.79 (8.263) | 6 (5) | **0.0067** (0.1423) |
| SW2_IS1.3 | *Dermacoccus* | 40.87 (28.93) | 8 (7) | **>0.0001 (0.0002)** |
| SO5_IS11 | *Curtobacterium* | 16.51 (13.30) | 7 (6) | **0.0209 (0.0385)** |

**Supplementary figure legends**

Figure S1. Examples of growth of antagonists alone (*Bacillus licheniformes*: **A** and *Pseudomonas monteilii*: **D**), co-culture with GT4_IS1 bacterial strain (**B** and **E**), and GT1_IS4 growth alone at the end of the bio-assays (28^th^ day).

Figure S2. Growth (± SE) of antagonists with different uropygial gland bacterial isolates for 28 days. Bacterial isolates represented with same color belong to the same genus and the control growth of antagonists are shown in black.

Figure S3. Growth (+ SE) of uropygial gland bacterial isolates with different antagonists for 28 days. Control growth of isolates is shown with black circles and connected with a black line.

Figure S4. Average growth ( ±SE) at day 28 of antagonists growing with bacterial isolates (black) compared to control growth (grey). Replicate numbers given within each bar, and asterisks indicate cases with significantly reduced growth compared to controls (Tables 2 and

Figure S5. Average growth on day 28 of uropygial bacterial isolates growing with different antagonists (black) compared to control growth alone (grey). Numbers within each bar represent the number of replicates, while asterisk indicate significantly reduced growth compared to controls from Table S6.
