# Supplementary material for: Great Tit (Parus major) Uropygial Gland Microbiomes and Their Potential Defensive Roles
Source: Front Microbiol. 2020 Jul 28;11:1735. doi: 10.3389/fmicb.2020.01735 (PMC7401573; doi:10.3389/fmicb.2020.01735)

A. *Bacillus licheniformis*

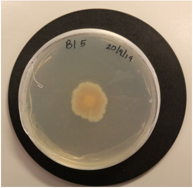

B. *Licheniformis* with GT4_IS1

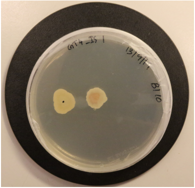

C. GT4_IS1 growing alone

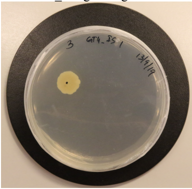

D. *Pseudomonas monteilii*

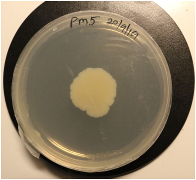

E. *P. montelii with* GT4_IS1

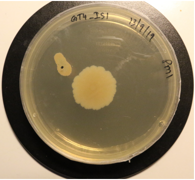

Supplement: Supplementary file 5 [file Image_1.pdf]
