# Supplementary material for: Great Tit (Parus major) Uropygial Gland Microbiomes and Their Potential Defensive Roles
Source: Front Microbiol. 2020 Jul 28;11:1735. doi: 10.3389/fmicb.2020.01735 (PMC7401573; doi:10.3389/fmicb.2020.01735)
Supplement: Supplementary file 6 [file Image_2.pdf]

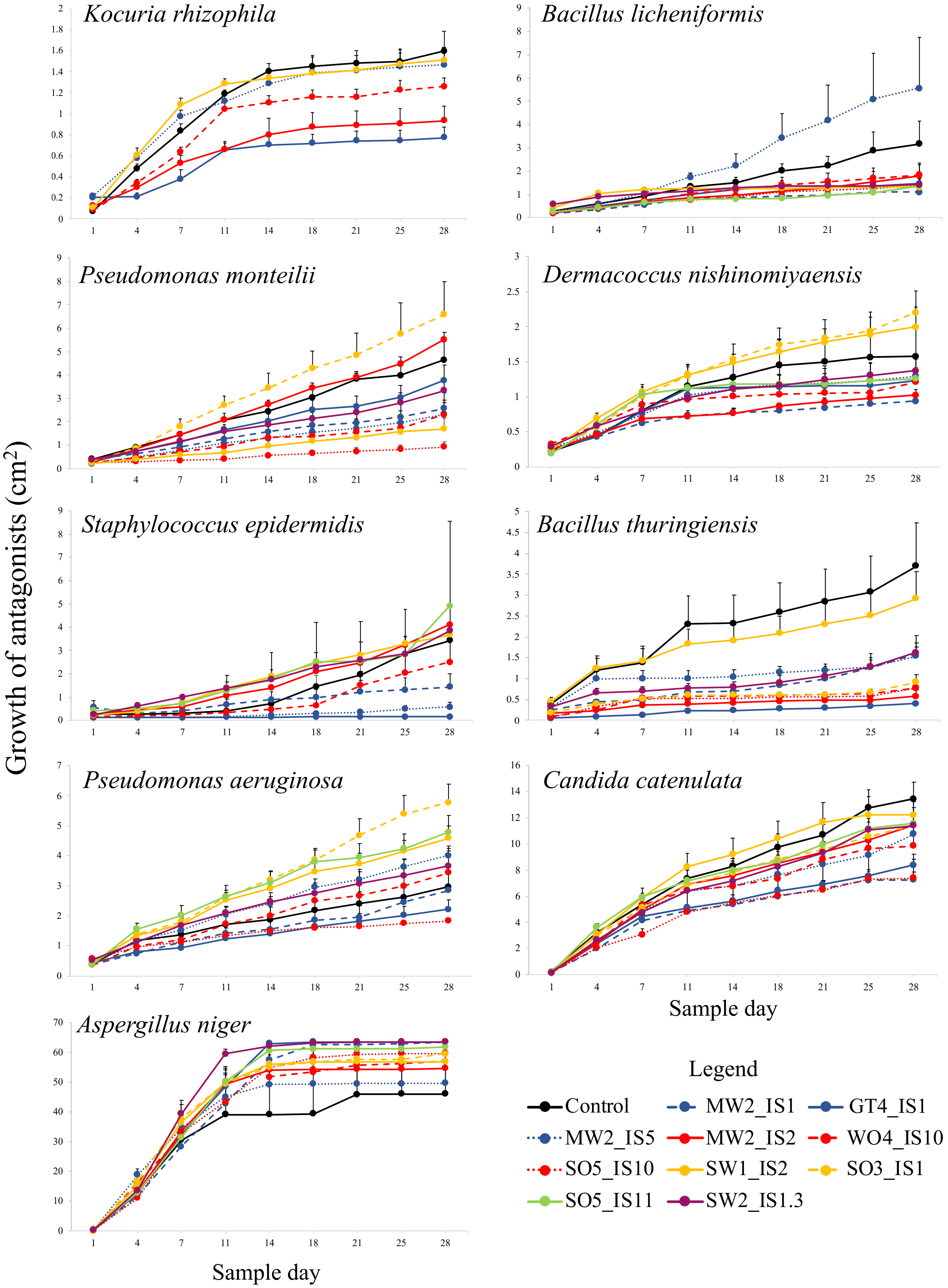

Kocuria rhizophila
Bacillus licheniformis
Pseudomonas monteilii
Dermacoccus nishinomiyaensis
Staphylococcus epidermidis
Bacillus thuringiensis
Pseudomonas aeruginosa
Candida catenulata
Aspergillus niger
Growth of antagonists (cm²)
Sample day
Legend
Control
MW2_IS1
GT4_IS1
MW2_IS5
MW2_IS2
WO4_IS10
SO5_IS10
SW1_IS2
SO3_IS1
SO5_IS11
SW2_IS1.3
