# Supplementary material for: Great Tit (Parus major) Uropygial Gland Microbiomes and Their Potential Defensive Roles
Source: Front Microbiol. 2020 Jul 28;11:1735. doi: 10.3389/fmicb.2020.01735 (PMC7401573; doi:10.3389/fmicb.2020.01735)
Supplement: Supplementary file 7 [file Image_3.pdf]

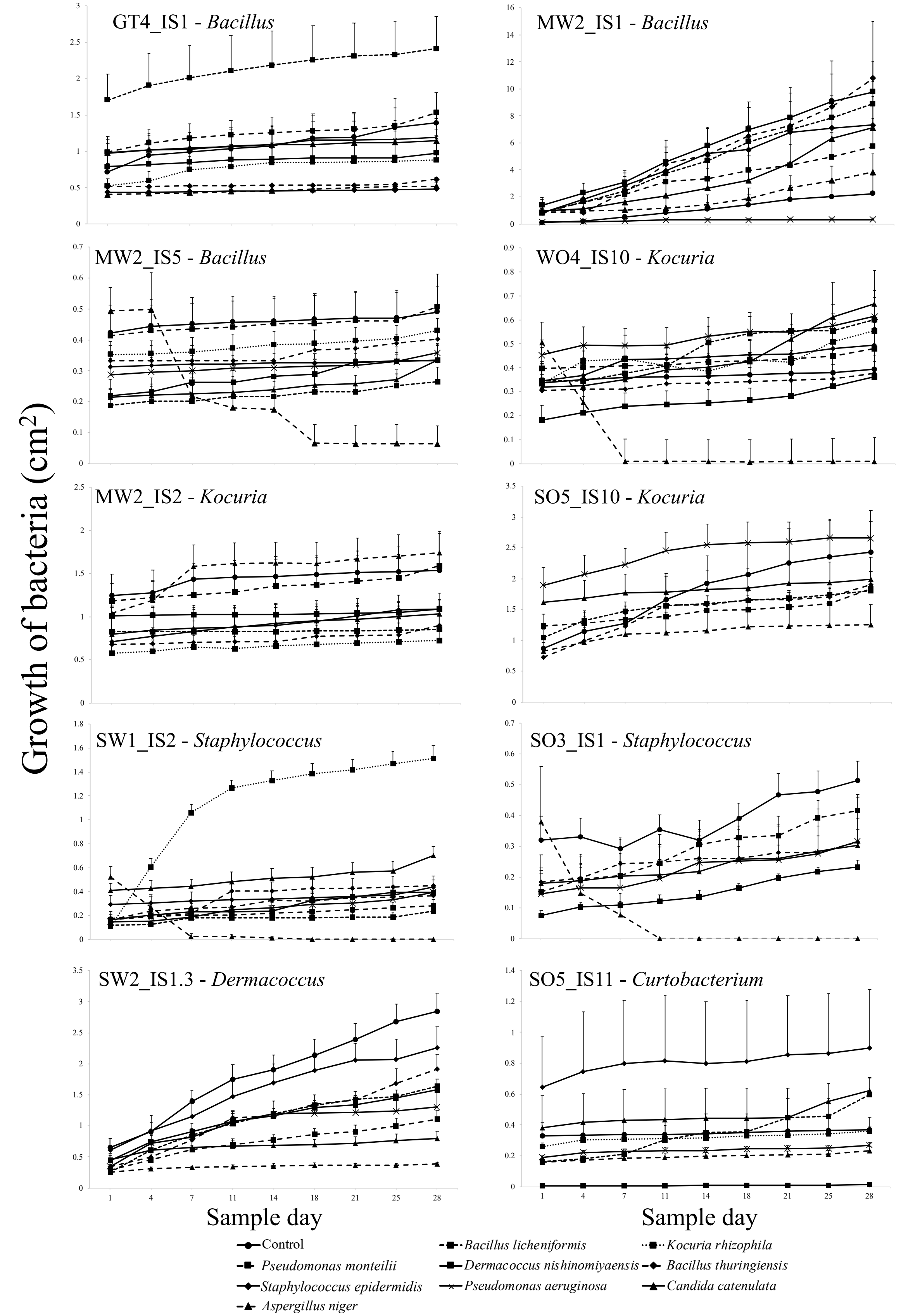
GT4_IS1 - Bacillus
MW2_IS1 - Bacillus
MW2_IS5 - Bacillus
WO4_IS10 - Kocuria
MW2_IS2 - Kocuria
SO5_IS10 - Kocuria
SW1_IS2 - Staphylococcus
SO3_IS1 - Staphylococcus
SW2_IS1.3 - Dermacoccus
SO5_IS11 - Curtobacterium
Growth of bacteria (cm2)
Sample day
Sample day
Control
Bacillus licheniformis
Kocuria rhizophila
Pseudomonas monteilii
Dermacoccus nishinomiyaensis
Bacillus thuringiensis
Staphylococcus epidermidis
Pseudomonas aeruginosa
Candida catenulata
Aspergillus niger
