# Supplementary figures and images for: Great Tit (Parus major) Uropygial Gland Microbiomes and Their Potential Defensive Roles
Source: Front Microbiol. 2020 Jul 28;11:1735. doi: 10.3389/fmicb.2020.01735 (PMC7401573; doi:10.3389/fmicb.2020.01735)

Growth of antagonists (cm<sup>2</sup>)

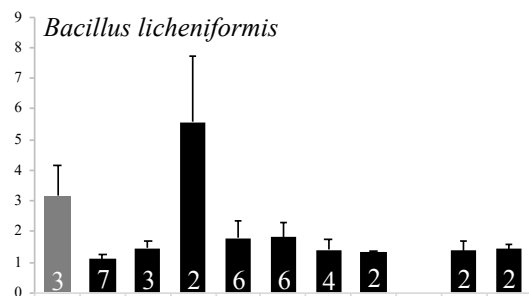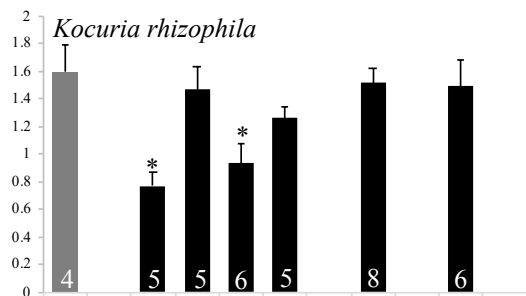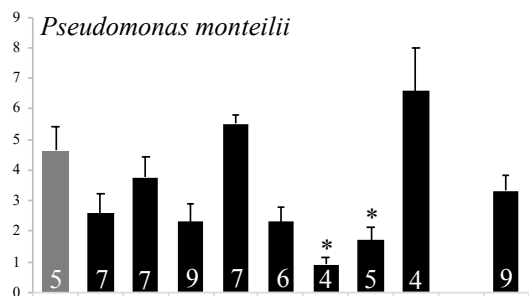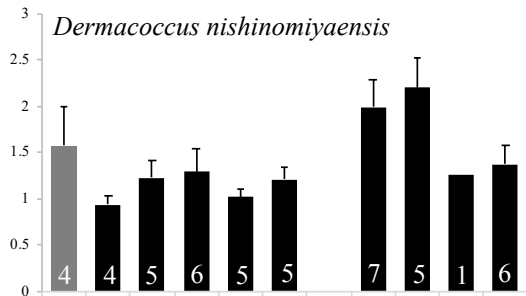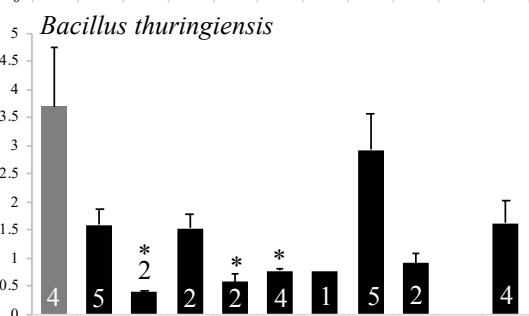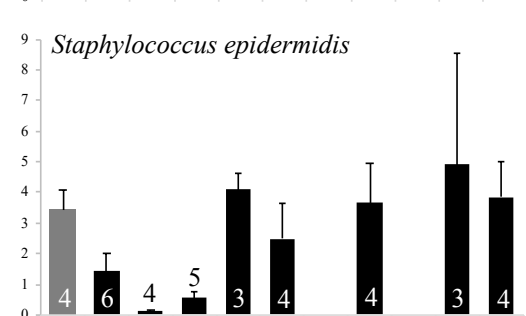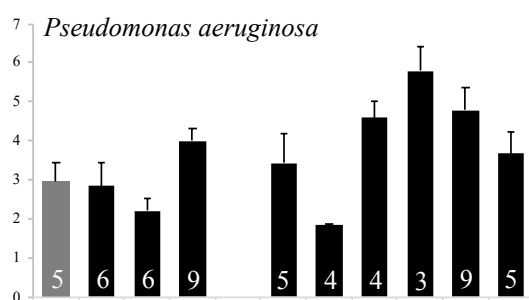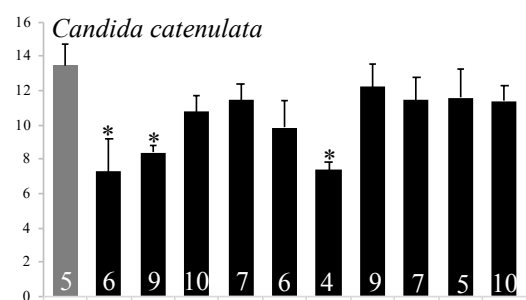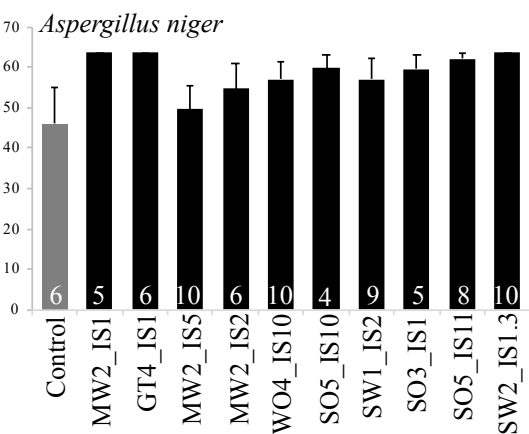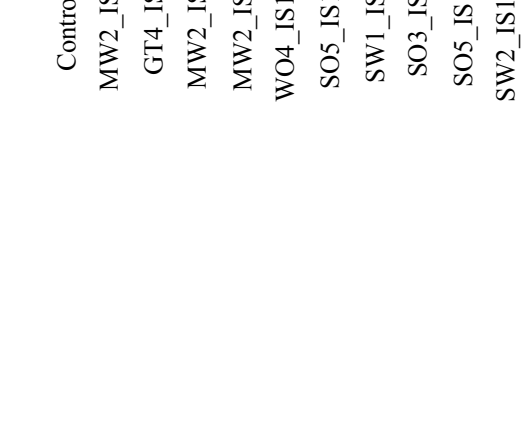

Supplement: Supplementary file 8 [file Image_4.pdf]

# Growth of bacteria (cm<sup>2</sup>)

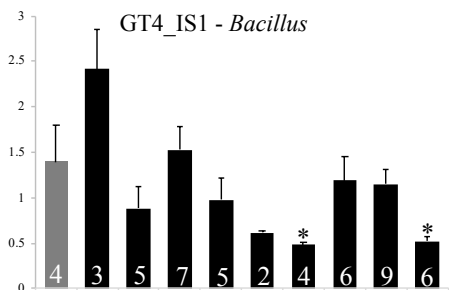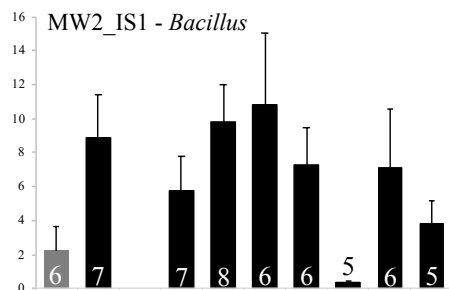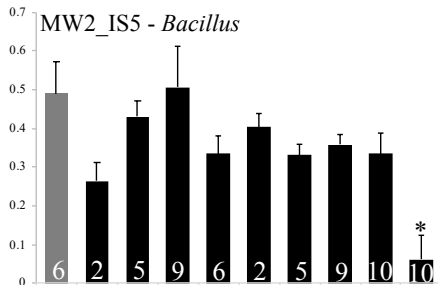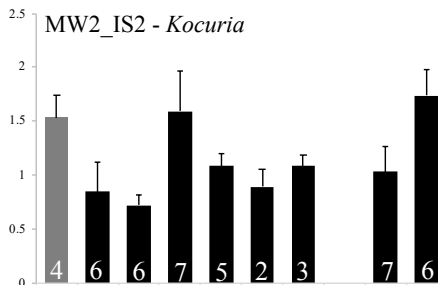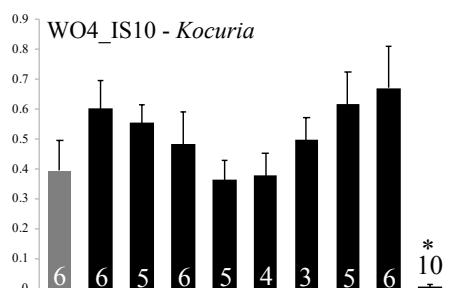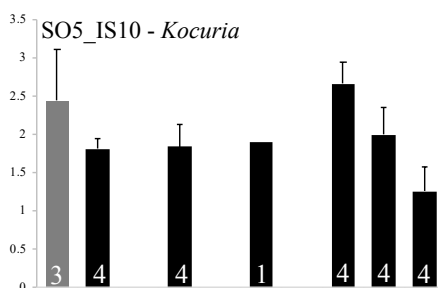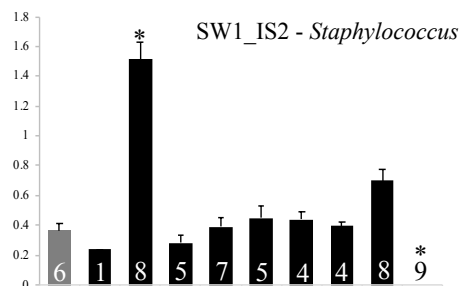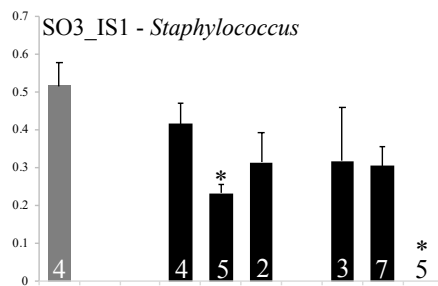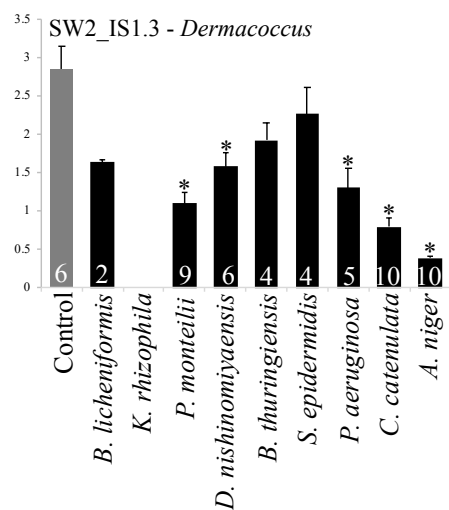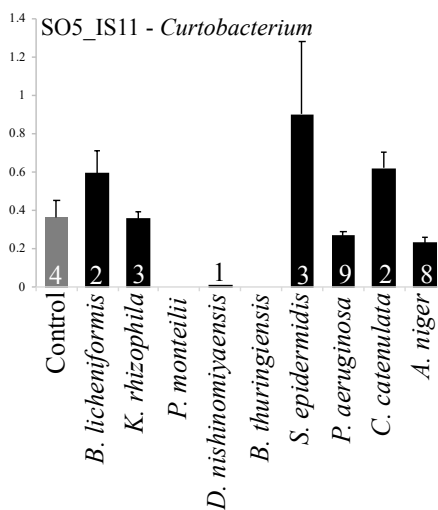

Supplement: Supplementary file 9 [file Image_5.pdf]
